# CD200 facilitates the isolation of corneal epithelial cells derived from human pluripotent stem cells

**DOI:** 10.1038/s41598-018-34845-2

**Published:** 2018-11-08

**Authors:** Ryuhei Hayashi, Yuki Ishikawa, Tomohiko Katayama, Andrew J. Quantock, Kohji Nishida

**Affiliations:** 1 Department of Stem Cells and Applied Medicine, Osaka University Graduate School of Medicine, Suita, Osaka 565-0871 Japan; 2 Department of Ophthalmology, Osaka University Graduate School of Medicine, Suita, Osaka 565-0871 Japan; 3 Structural Biophysics Group, School of Optometry and Vision Sciences, College of Biomedical and Life Sciences, Cardiff University, Cardiff, CF24 4HQ Wales UK

## Abstract

The *in vitro* induction of corneal epithelial cells (CECs) from human induced pluripotent stem cells (iPSCs) represents a new strategy for obtaining CE stem/progenitor cells for the surgical reconstruction of a diseased or injured ocular surface. The clinical promise of this strategy is considerable, but if the approaches’ potential is to be realised, robust methods for the purification of iPSC-derived CE lineage cells need to be developed to avoid contamination with other cells that may carry the risk of unwanted side effects, such as tumorigenesis. Experiments conducted here revealed that during CEC isolation, CD200-negative selection using a cell sorter considerably reduced the contamination of the cell population with various non-CECs compared with what could be achieved using TRA-1-60, a conventional negative marker for CECs. Furthermore, CD200-negative sorting did not affect the yield of CECs nor that of their stem/progenitor cells. Single-cell gene expression analysis for CEC sheets obtained using CD200-negative sorting showed that all analysed cells were CE-lineage cells, expressing *PAX6*, *delta-N p63*, and *E-cadherin*. Non-CECs, on the other hand, expressed non-CEC genes such as *FGFR1* and *RPE65*. CD200, thus, represents a robust negative marker for purification of induced CE lineage cells, which is expressed by undifferentiated iPSCs and non-CECs, including iPSC-derived neural and retinal cells.

## Introduction

Human pluripotent stem cells (PSCs) represent a promising cell source for regenerative therapy in several disease states^1,2^. To make this promise a reality, the development of tissue-specific, target-cell differentiation strategies is essential. Methods for the differentiation of various kinds of tissues or cells, including the retina and retinal pigment epithelial cells, neural stem cells, dopaminergic neurons, cardiac muscle cells, and corneal cells have all been reported^3–7^. However, methodologies for the differentiation of PSCs–including induced PSCs (iPSCs) and embryonic stem cells – often lead to the induction, not only of the intended target cells, but of other cell types too. This is a problem for regenerative therapies using PSC-derived cells because undifferentiated PSCs that remain in the expanded cell population or PSCs that differentiate down pathways other than the intended one carry the risk of serious side effects and these include potential teratoma formation. To obtain a broadly homogeneous population of cultivated PSC-derived cells, procedures using specific culture conditions^8,9^, cell sorting using cell surface markers^10–12^, and colony pick-up methods^3^ have been reported. Of these, cell sorting likely has the greatest potential to obtain a pure population of target cells, but, crucially, the purity of the cell culture largely depends on the specificity of cell surface makers used for cell sorting.

Previously, we reported a method for the induction of the primordial cells of the eye, (including corneal epithelial cells (CECs)) from human iPSCs, which we termed the self-formed ectodermal autonomous multi-zone (SEAM) method^13^. Using this method, a colony of cells could be formed that contained up to four different concentric zones formed of cells with distinct morphologies and immunostaining characteristics. In a number of respects the SEAM colony mimicked ocular development *in vivo*. For example, neuronal-like cells appear in in the innermost zone 1 of the SEAM, retinal-like or neural crest-like cells in the more peripheral zone 2, cells resembling CE primordial cells (i.e. CE stem/progenitor cells) in zone 3, and non-ocular epithelial-like cells in the outermost SEAM zone 4. This diversity with multiple cell lineages in the same culture, however, means that the differentiation efficiency of CE lineage cells is not very high^14^. Nevertheless, sufficient numbers of CE stem/progenitor cells could be obtained and expanded into functional epithelial multi-layers via our use of the cell surface markers SSEA-4 and ITGB4 as positive markers of CE lineage cells in conjunction with TRA-1-60 as a negative marker. Using this approach, we were able to isolate CECs, including their stem/progenitor cells, from the differentiated iPSCs^13,14^. Although this technique enabled isolation of CE lineage cells, they were not sufficiently pure, prompting the current investigation to investigate ways of removing untargeted cells and enhancing the purity of CECs induced from SEAMs using a cell surface marker. To achieve this we screened cell surface markers that can enhance the purity of CECs by cell sorting and identified CD200 as a new and effective negative marker to remove non-target cells from differentiated human iPSC cultures, permitting superior purification of CE lineage cells.

## Results

### CEC induction and isolation from human iPSCs

**Figure 1:**
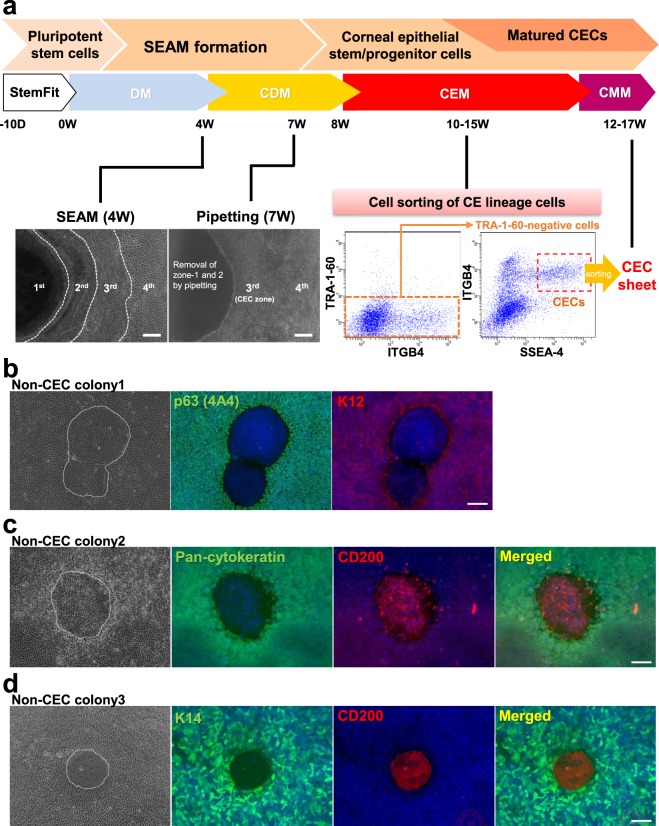
SEAM formation and the insolation of human iPSC-derived CE lineage cells. (**a**) Schematic representation of the procedure used for the generation of a SEAM from human iPSCs and the subsequent isolation of CE lineage cells. CECs appeared in zone 3 of the SEAM at 3 to 4 weeks after the initiation of differentiation. Non-epithelial cellular zones 1 and 2 were removed by pipetting at week 7. At weeks 10 to 15, iPSC-derived CE lineage cells were isolated by cell sorting using anti-SSEA-4, anti-ITGB4, and anti-TRA-1-60 antibodies. Bars, 200 μm. (**b**) Representative immunostaining images showing how the expression of corneal epithelial markers p63 (green) and K12 (red) in non-CEC colony (no. 1) emerged in the iPSC-derived CEC sheets. (**c**,**d**) Representative immunostaining images of epithelial cell markers, pan-cytokeratin or K14 (green) and CD200 (red), in non-CEC colonies (nos 2, 3) in the iPSC-derived CEC sheets. Nuclei, blue. Scale bars, 50 μm.

CEC differentiation from human iPSCs via SEAM formation was conducted according to our previously reported methods^14^, and by weeks 3 to 4 of differentiation SEAMs comprising typical cellular multi-zones were formed (Figs 1a and S1a). After removing the inner non-epithelial zones of the SEAM (i.e. zones 1 and 2), CE lineage cells differentiated in zone 3 were isolated by cell sorting at weeks 10 to 15 after initiation of differentiation. The majority of cells isolated using the conventional combination of cell surface markers, including TRA-1-60, SSEA-4, and ITGB4, were found to be CE lineage cells. Nevertheless, several non-CEC colonies comprising cells not expressing stratified epithelial cells and CEC markers, such as p63 and K12, were obtained also (Fig. 1b). Interestingly, our analyses revealed that many of these non-epithelial cells, which showed negative staining for pan-cytokeratin and K14, expressed CD200 (Fig. 1c,d)

### CD200 expression during human iPSC differentiation

**Figure 2:**
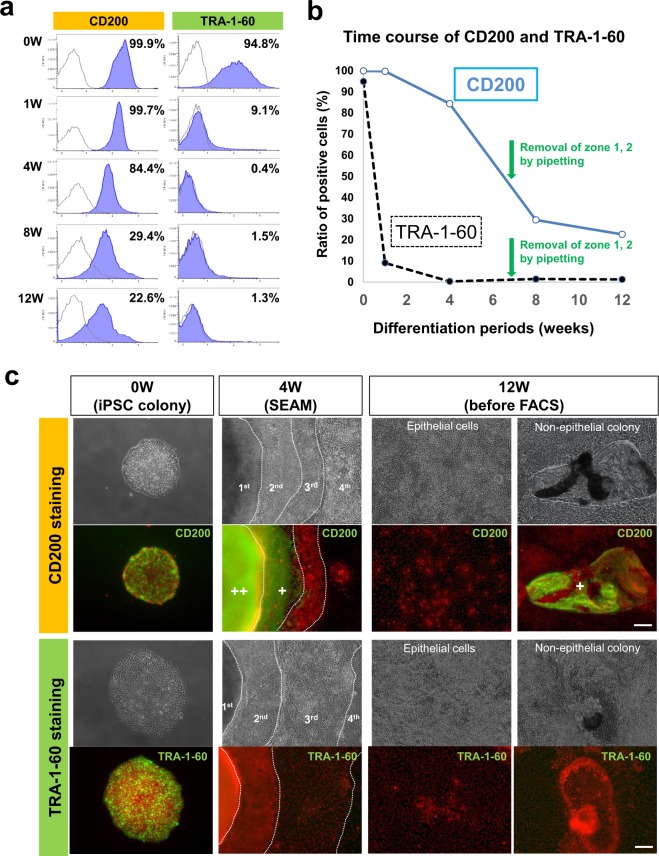
CD200 and TRA-1-60 expression during the differentiation of human iPSCs. (**a**) Flow cytometric results showing CD200 and TRA-1-60 expression on iPSCs at weeks 0, 1, 4, 8, and 12 after the initiation of differentiation. (**b**) CD200- and TRA-1-60-positive to negative cell ratio changes with the culture time. (**c**) Representative CD200 and TRA-1-60 (green) immunostaining images of undifferentiated iPSCs (0 week) and differentiated iPSCs at 4 weeks (4 w, SEAM) and 12 weeks (12 w, before cell sorting). Nuclei, red. Scale bars, 50 μm.

Based on the identification of CD200 expressed in non-CEC cultures, we sought to explore its use as a potential marker for CE lineage cell isolation from human iPSCs differentiated using the SEAM method. Initially, the expression of CD200 with time during iPSC differentiation was examined (Fig. 2a). Flow cytometry demonstrated that CD200 is highly expressed by all undifferentiated iPSCs before differentiation and by 80% of iPSCs even after 6 weeks of differentiation. After removing non-epithelial-like cellular zones from the SEAM at week 7, CD200 expression decreased considerably, but remained at around 20% even at week 12 (Fig. 2b). In contrast, TRA-1-60 expression was below 10% immediately after the early differentiation steps, at week 1, ultimately dropping to approximately 1% at week 12 (Fig. 2b). Immunostaining indicated that CD200 was strongly expressed by all undifferentiated iPSCs and non-epithelial cells (i.e. such as those in zones 1 and 2 of the SEAM at week 4), but was only faintly expressed in zone 3 (Fig. 2c). At 12 weeks of culture, the majority of differentiated CEC-like cells showed no CD200 expression, while some non-CEC-like cells did. The expression of TRA-1-60 was limited to the undifferentiated iPSCs, with negligible expression of this molecule during the differentiation (Fig. 2c).

### Isolation and characterization of human iPSC-derived CE lineage cells using CD200

**Figure 3:**
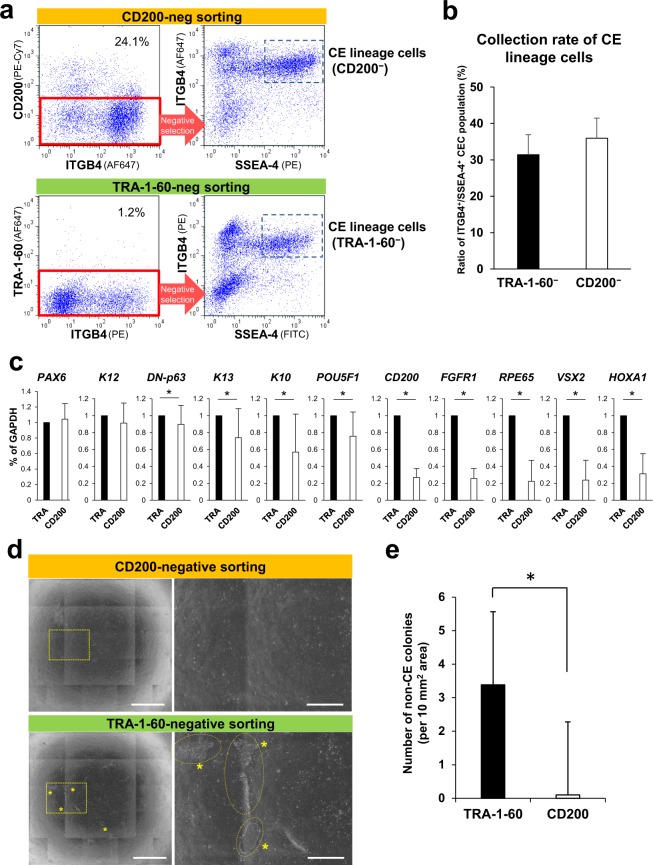
Isolation of human iPSC-derived CE lineage cells by CD200-negative sorting. (**a**) Flow cytometric analyses of SSEA-4, ITGB4, and CD200 (upper panels) or TRA-1-60 (lower panels) expression by differentiated iPSCs at weeks 10–15 of differentiation. ITGB4^+^/SSEA-4^+^ cells were considered to be CE lineage cells obtained from CD200- or TRA-1-60-negative populations, respectively. (**b**) Collection rate of iPSC-derived CE lineage cell population (ITGB4^+^/SSEA-4^+^) obtained by negative sorting procedures. Error bars, SD (n = 6). (**c**) Expression of corneal and non-target cell-related genes by the isolated iPSC-derived CE-like cells. Relative expression levels were set as 1 in TRA-1-60-negative samples (TRA). Error bars, SD (n = 9); *p < 0.05. (**d**) Representative phase-contrast images showing the cultivated iPSC-derived CE-like cells isolated by CD200- (upper panels) or TRA-1-60-negative sorting (lower panels). Right: magnified areas shown in the left panels. Asterisks: non-target colonies. Scale bars: 2 mm (left), 500 μm (right). (**e**) Numbers of non-target colonies in the cultivated iPSC-derived CE-like cells. Error bars, SD (n = 4); *p < 0.05.

To investigate CD200 as a potential negative marker for CE lineage cells, cell sorting using an anti-CD200 antibody was performed. After 10 to 15 weeks of differentiation, iPSC-derived CE lineage cells were isolated by using anti-ITGB4, anti-SSEA-4, and anti-CD200 or anti-TRA-1-60 antibodies (Fig. 3a). The collection rate for CE lineage cells, defined as ITGB4^+^/SSEA-4^+^ cells, was 36.9 ± 5.2% (n = 6) for CD200-negative cells, and 33.2 ± 5.2% (n = 6) for TRA-1-60-negative cells, with no statistically significant difference between both methods (Fig. 3b). Isolated cells were analysed by qPCR to determine the potential contamination of the sorted population (Figs 3c and S1b) and this revealed a significant decrease in the expression of non-target markers, including *FGFR1* (expressed by non-epithelial cells including fibroblasts, neuronal cells, and human iPSCs)^15–17^, *RPE65* (retinal pigment epithelial cell)^18^, *HOXs* (non-ocular trunk cells)^19^, *COL1A1* and *A2* (fibroblasts)^20^, *PDGFRA* and *PDGFRB* (mesodermal and vascular cells)^21,22^ and *CD200* by the isolated cells selected for their lack of CD200 expression. In contrast, no changes in the expression of CEC-associated genes, such as *PAX6* and *K12*, were observed. Non-epithelial-like colonies developed from the sorted CE lineage cell samples indicated that there were significantly fewer non-CECs in CD200-negatively sorted cells compared with the TRA-1-60-negative samples (Fig. 3d). Phase-contrast analyses demonstrated that after CD200-negative sorting, almost no recognizable non-target colonies developed from the CD200-negative iPSC-derived CE lineage cells, unlike the situation using samples obtained by TRA-1-60-negative sorting (Fig. 3e).

### CE stem/progenitor cells and CD200 *in vitro* and *in vivo*

**Figure 4:**
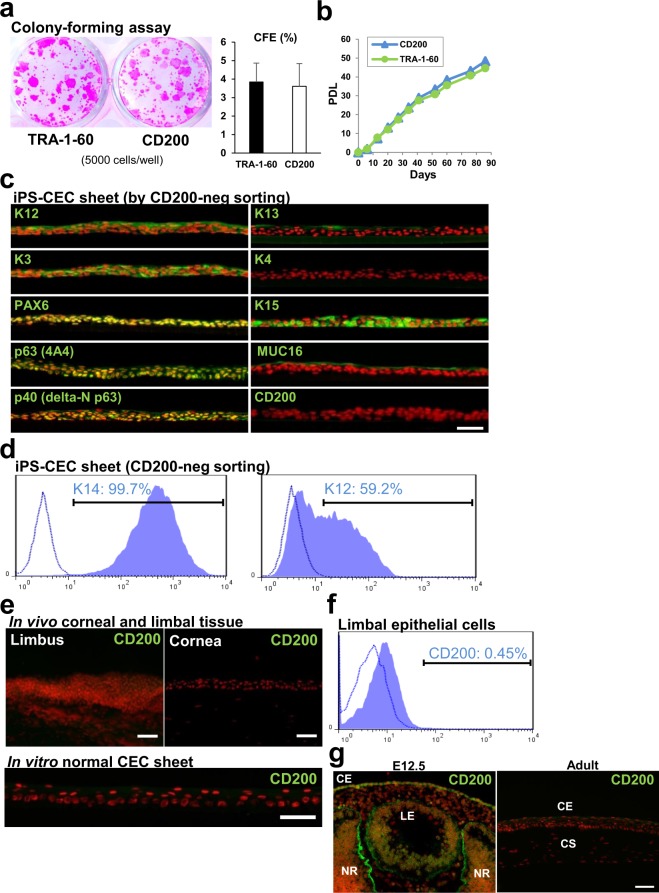
CFE of human iPSC-derived CE lineage cells, and CD200 expression in corneal tissue. (**a**) CFA analysis performed using iPSC-derived CE lineage cells isolated by CD200- or TRA-1-60-negative sorting (5000 cells/well). Right, CFEs of both samples. Error bars, SD (n = 4). (**b**) Serial cell passaging assay for iPSC-derived CE lineage cells isolated by CD200- or TRA-1-60-negative sorting. PDL; Population doubling level. (**c**) Representative immunostaining images showing corneal-related maker and CD200 expression (green) by iPSC-derived CEC sheets obtained by CD200-negative sorting. Nuclei, red. Scale bar, 50 μm. (**d**) Flow cytometric analysis of K14 and K12 expression by iPSC-derived CE lineage cells obtained by CD200-negative sorting. (**e**) Representative CD200 immunostaining images showing its non-expression by the corneal and limbal tissues as well as cultivated limbal epithelial cell sheet derived from human limbal tissue. Nuclei, red. Scale bars, 50 μm. (**f**) Flow cytometric analysis of CD200 expression by limbal epithelial cells. (**g**) Immunostaining of CD200 (green) in murine embryonic (E12.5) and adult eyes. Nuclei, red. CE; Corneal epithelium, CS; Corneal stroma, LE; Lens, NR; Neuro-retina. Scale bar, 50 μm.

To assess the presence of CE stem/progenitor cells in each cell population, colony-forming assay (CFA) experiments were performed. These disclosed no differences in colony-forming efficiency (CFE) between CD200- and TRA-1-60-negative sorting samples (3.6 ± 1.2 *vs*. 3.8 ± 1.0%, respectively; n = 4) (Fig. 4a). To evaluate the proliferative potential further, a serial cell passaging assay was conducted (Fig. 4b). The results showed that iPSC-derived CE stem/progenitor cells isolated by both CD200 and TRA-1-60-negative sorting exhibited similar levels of population doubling. These data indicated that CD200-negative sorting successfully allowed the harvesting of not only differentiated CECs, but of stem/progenitor cells too. Moreover, iPSC-derived CE stem/progenitor cells isolated by CD200-negative sorting became stratified and were able to successfully form an epithelial cell sheet. This cell sheet expressed CEC-specific makers, including K12, PAX6, K3, and MUC16, but showed no expression of CD200 or conjunctival epithelial markers, K4 and K13 (Fig. 4c). K15, p63 and p40 (delta-N p63 isoform), stratified epithelial stem/progenitor cell markers, were expressed in some basal cells (Fig. 4c). Anti-K3 and K12 antibodies clearly stained the intermediate filament-like structure in the cultivating CECs at day 7, although there were still many CE stem/progenitor-like cells which did not express K3 or K12 at this point (Supplementary Fig. S1c). Flow cytometric analysis of K14 and K12 expression demonstrated that CEC sheets comprised 99% K14^+^ stratified epithelial cells and approximately 60% K12^+^ differentiated CECs (Figs 4d and S1d). Immunostaining of corneal tissue revealed no CD200 expression in the corneal epithelium, including the limbal region at the edge of the cornea where epithelial stem cells are believed to reside (Fig. 4e). Similarly, limbal epithelial cells maintained *in vitro* showed no CD200 expression (Fig. 4e), and flow cytometric analyses demonstrated minimal CD200 expression in human limbal epithelium *in vivo* (Fig. 4f). We further examined CD200 expression in murine embryonic and adult eyes (Fig. 4g). Immunostaining data showed that CD200 was expressed in ocular tissues, including corneal epithelium and retinal cells in the E12.5 embryonic eye. In contrast, its expression in the corneal epithelium was lost in the adult mouse eye.

### Single-cell gene expression analysis of human iPSC-derived CE lineage cells isolated using CD200-negative sorting

**Figure 5:**
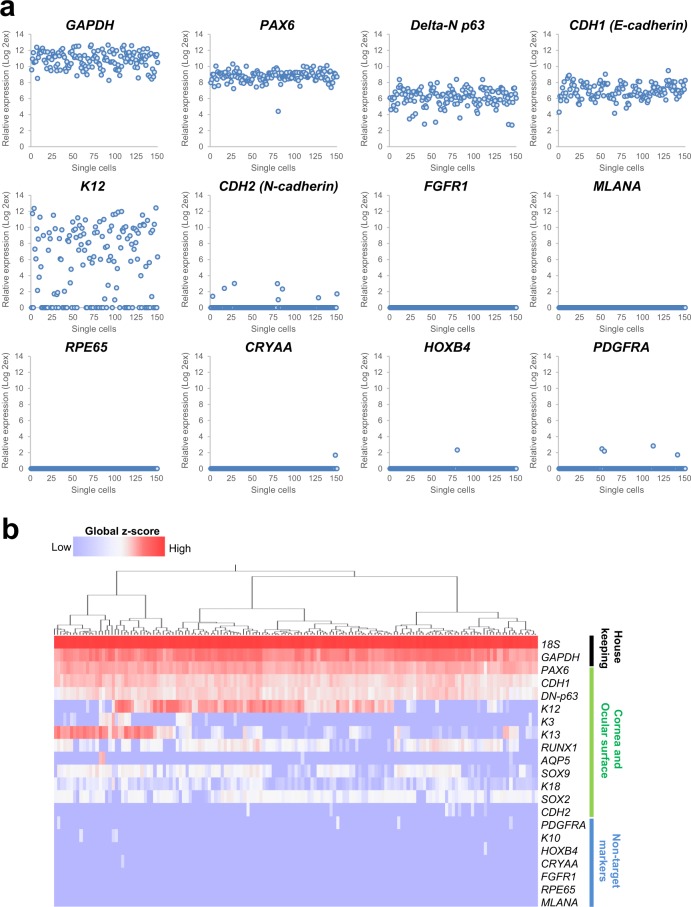
Single-cell gene expression analysis of iPSC-derived CEC sheet. (**a**) Representative data showing the expression of corneal and non-target cell-associated genes by the iPSC-derived CE-like single cells obtained using CD200-negative sorting. A total of 151 cells were analysed. (**b**) Heat map obtained using hierarchical cluster analysis based on the single-cell gene expression results.

Single-cell gene expression analysis of cells isolated using CD200-negative sorting revealed the expression of 21 housekeeping, CEC-, and non-target cell-related genes as determined using 151 iPSC-derived CE-like cells. The analysis revealed that all cells analysed exhibited a *PAX6*^+^/*delta-N p63*^+^/*CDH1* (*E-cadherin)*^+^ CEC phenotype (Fig. 5a,b). No expression of *FGFR1* (a non-epithelial cell marker), *RPE65* or *MLANA* (melanocyte markers)^23^ was observed, along with negligible expression of *CRYAA* (lens cell marker, 1/151 cells)^13^, *HOXB4* (non-ocular epithelial cell marker, 1/151 cells), *K10* (keratinocyte marker, 3/151 cells), and *PDGFRA* (mesodermal and mesenchymal marker, 4/151 cells)^21^. Approximately 67% of the cells were *K12*^+^ (102/151 cells), while 5.3% were *CDH2* (*N-cadherin*)^+^, indicating a CE stem/progenitor cell phenotype (8/151 cells)^24^.

## Discussion

Previous work has used the SEAM method to induce the formation of ocular primordia from human iPSCs^13,14^. This results in the generation of a concentric cellular multi-zone in which cells in SEAM zone 3 possess the characteristics of CE lineage cells. But, because a range of cell types exists within a single colony, cell sorting is required to isolate cells with the desired genotype/phenotype, and non-contamination with other cell types is a priority if the intended use is for clinical purposes. In our previous research, to discard undifferentiated iPSCs we used TRA-1-60 as a PSC marker. However, the expression of this molecule decreases considerably before cell sorting, so it is not the most effective agent for the removal of non-target cells and a novel more effective cell surface maker is required.

We performed screening for a cell surface marker to remove non-targeted cells and enhance the purity of CECs via the use of previously used microarray databases (GSE73971)^13^. We searched for negative markers that are expressed in undifferentiated iPSCs and in cells other than CECs, but which were negative for CECs. This analysis identified *CD200*, *FGFR1*, and *PDGFRB* (Supplementary Fig. S2a), and among these three markers only anti-CD200 antibody (OX-104, commercially available) stained human iPSCs (but not CECs) and could detect an extracellular region of the antigen in flow cytometry as shown in Figs 1c,d, 2 and 4f.

CD200 is a glycoprotein widely expressed in somatic cells. It is a marker of breast cancer, leukaemia, and colon cancer cells, as well as being a PSC marker^12,25,26^. Here, we demonstrate that CD200 is uniformly expressed by undifferentiated human iPSCs, and during differentiation, CD200 expression is sustained by more than 80% of iPSCs, even after four weeks of culture. Its expression is thus more stable than that of TRA-1-60, which drops considerably in the early differentiation period. Furthermore, by the end of the differentiation culture, TRA-1-60 expression had become drastically decreased to around 1% of cells. In contrast, after the same differentiation period, CD200 was still maintained in approximately 20% of differentiated iPSCs. These findings show that TRA-1-60 is specific to the undifferentiated state of PSCs, but is no longer expressed in differentiating iPSCs, which probably maintain multi-lineage differentiation potential as well as tumour formation capability. Therefore, after the differentiation culture, TRA-1-60 is no longer useful in removing these differentiating non-CECs because the expression would have already been lost. In contrast to TRA-1-60, CD200 expression was maintained in the differentiating iPSCs even after 12 weeks, implying that CD200 is broadly expressed not only in undifferentiated iPSCs, but also in differentiating or differentiated iPSCs other than CE lineage cells. The immunostaining data clearly indicates that during differentiation culture, CD200 expression was particularly well maintained in non-CEC zones such as zone 1 and 2, indicating that CD200 could be a positive selector of various non-CEC lineages obtained from differentiated iPSCs.

Regarding ITGB4 and SSEA-4 used in combination with CD200, each solo marker cannot specifically select corneal epithelial cells although we previously found that corneal and limbal epithelial cells express both ITGB4 and SSEA-4. In fact, previous reports indicated that each marker is expressed even in some non-epithelial cells in culture^27,28^ and our present result further indicates that ITGB4^+^/SSEA-4^+^ cells without CD200-negative sorting contained small numbers of non-epithelial cells. Collectively, these facts suggest that CD200 possibly compensates for the limitations when using ITGB4 and SSEA-4 as selective markers for CE lineage cells.

The findings of a gene expression analysis also suggest that CD200 can be used to effectively remove non-CECs such as neuronal cells, fibroblasts, retinal cells, and keratinocytes from the SEAM colony. The most pronounced CD200-instigated negative selection was discovered to be for *FGFR1*-expressing cells. FGFR1 is expressed by PSCs and by a range of non-epithelial cells, including neuronal cells and fibroblasts, suggesting that it may be a useful indicator of possible contamination of non-stratified epithelial cells. FGFR1 is a cell-surface protein, but when we assessed its potential use for the negative marker for selection of CECs using commercially available anti-FGFR1 antibodies, it transpired that the cell sorting we performed was not successful. Perhaps this is because extracellular regions of FGFR1 protein were digested by enzymatic treatment at cell harvesting. In contrast to that of the non-CEC genes, no remarkable changes in the expression of CEC-associated makers, including *PAX6*, *delta-N p63*, and *K12*, were observed. Flow cytometry and CFA results, moreover, disclosed no difference in the yields of CECs and their stem/progenitor cells obtained by CD200- or TRA-1-60-negative sorting.

As shown here, CD200 is primarily expressed by non-epithelial cells, although during differentiation culture some transient expression was detected in ITGB4^+^ epithelial cells (Supplementary Fig. S1e). This expression, however, is down-regulated by week 12, and in adult corneal tissue and in limbal epithelial cells grown *in vitro* almost no CD200 expression was detected (Fig. 4e,f). Moreover, we confirmed that CD200 expression was found only in developmental CECs, not in adult CECs (Fig. 4g). These data suggest that CECs transiently express CD200 during their development, but not when fully differentiated. Previous reports indicated that CD200 represents a hair follicle stem cell marker^29,30^, suggesting that the CD200^+^ iPSC-derived epithelial fraction might contain other types of epithelial cells, including hair follicle stem cells.

Single-cell gene expression analysis results showed that all analysed cells (n = 151) isolated by CD200-negative selection showed *PAX6*, *delta-N p63*, and *E-cadherin* expression; non-epithelial cell markers, such as *FGFR1*, *RPE65*, and *MLANA*, on the other hand, were essentially not expressed. Other non-target cell-related genes were slightly expressed by a very low number of cells in the isolated population. Use of anti-CD200 antibodies, therefore, allows us to eliminate non-target cells and enrich CECs in CD200-negatively isolated cell populations, creating a high purity iPSC-derived CE lineage cell population. In the current experiments a small number of the analysed CE lineage cells (8/151) expressed the CE stem/progenitor cell specific marker, *N-cadherin*, with majority of these cells (6/8) not expressing corneal differentiation markers, *K3* and *K12*, nor non-CEC makers, such as *FGFR1*, *CRYAA*, and *PDGFRA*. This suggests that a number of undifferentiated CE stem/progenitor cells can be found among CD200-negatively isolated CE lineage cells. Single-cell gene expression analysis and immunostaining for iPSC-derived CEC sheets demonstrated different expression patterns between K3 and K12, even though they should form a pair. Our previous data indicated that long-term culture (i.e. more than 10 weeks) promoted CEC maturation–as evidenced by an enhancement of stratification and keratin expression–over and above that seen within shorter culture periods (3 weeks)^14^. Additionally, a previous report has suggested that K3 and K12 expression in embryonic cornea is not coincident^31^, whilst another report has pointed to a differential regulation of K3 and K12 expression by PAX6 isoforms^32^. These reports of a discordant expression of K3 and K12 during developmental, may indicate the possibility of an incomplete maturation of our CECs *in vitro* due to an insufficient culture period.

The corneal epithelium is maintained by stem cells located in the basal region of the limbal epithelium at the edge of the cornea^33^. Regenerative medicine involving the transplantation of iPSC-derived CEC sheets including stem/progenitor cells presents a promising strategy for the treatment of stem cell deficiencies caused by CE dysfunction. However, to be clinically applicable, contamination with non-target cells must be avoided because of the potential of unwanted side effects. The results obtained here show that CD200 is preferentially expressed by undifferentiated iPSCs and non-CECs, identifying it as a robust negative selector of hiPSC-derived CE lineage cells. CD200 is also a cancer-associated marker^31–33^, thus, CD200-negative sorting may also limit the risk of tumorigenesis. CD200-negative sorting is therefore viewed as a favoured approach for first-in-human clinical trials of corneal epithelial regenerative medicine.

## Methods

All experiments were performed in accordance with the relevant institutional and national guidelines and regulations.

### Human iPSCs

The human iPSC line 201B7 was purchased from RIKEN Bio Resource Center (Tsukuba, Japan). After passaging the cells on a mitomycin C (MMC)-treated mouse embryonic fibroblast (MEF) feeder layer at least three times, they were cultivated in a feeder-free system using Laminin-511 E8 fragments (LN511E8; Nippi, Tokyo, Japan) and serum-free medium (StemFit; Ajinomoto, Tokyo, Japan) for 7 days^34,35^. All experiments using recombinant DNA were approved by the Recombinant DNA Committee of Osaka University and were performed according to our institutional guidelines.

### Differentiation of cells using the SEAM method

The differentiation of ocular cells from human iPSCs was performed according to the previously reported method^14^. Briefly, human iPSCs were seeded onto LN511E8-coated (0.5 μg/cm^2^) plates and cultivated in StemFit medium for 10 days, after which the medium was changed to serum-free differentiation medium [DM; GMEM (Life Technologies, Carlsbad, CA, USA) supplemented with 10% knockout serum replacement (KSR; Life Technologies), 1 mM sodium pyruvate (Life Technologies), 0.1 mM non-essential amino acids (Life Technologies), 2 mM L-glutamine (Life Technologies), 1% Penicillin-Streptomycin solution (Life Technologies) and 55 μM 2-mercaptoethanol (Life Technologies) or monothioglycerol (Wako, Osaka, Japan)]^13,14^. After 4 weeks, the medium was replaced with corneal differentiation medium (CDM) supplemented with 10–20 ng/mL KGF (Wako) and 10 μM Y-27632 (Wako) and cultured for an additional 4 weeks. Non-epithelial cells were removed from the SEAM by pipetting at around week 7 after initiation of differentiation. Following this, the medium was replaced with corneal epithelium maintenance medium (CEM) containing 2% B27 supplement (Life Technologies), 10–20 ng/mL KGF, and 10 μM Y-27632 at week 8, and the plates were incubated for an additional 2 to 7 weeks (i.e. 10–15 weeks in total).

### Flow cytometry and cell sorting

Flow cytometry and cell sorting were performed as previously reported^14^. After 10 to 15 weeks in the culture, differentiated iPSCs were harvested by Accutase (Life Technologies) and re-suspended in ice-cold KCM medium. The cells were stained with Alexa-Fluor 647 (AF647)- or PE-conjugated anti-ITGB4 (58XB4, BD Biosciences, San Jose, CA, USA or Biolegend, San Diego, CA, respectively), PE- or FITC-conjugated anti-SSEA-4 (MC813-70, Biolegend), and PE-Cy7-conjugated anti-CD200 (OX-104, BD Biosciences) or AF647-conjugated anti-TRA-1-60 (TRA-1-60-R, Biolegend) antibodies for 1 h on ice. In addition, the isotype control antibodies corresponding to each antibody were used. Flow cytometry and cell sorting were performed with a FACS AriaII (BD Biosciences) according to the manufacturer’s instructions. CE lineage cells were isolated as a fraction of ITGB4^+^/SSEA-4^+^/CD200^−^ or ITGB4^+^/SSEA-4^+^/TRA-1-60^−^ cells. Data were analysed using the BD FACSDiva Software (BD Biosciences) and the FlowJo software programs (TreeStar, San Carlos, CA, USA).

### CFA and cell sheet generation

For the CFA, CE lineage cells isolated by cell sorting were seeded onto MMC-treated NIH-3T3 feeder cells at the cell density of 2000–5000 cells/well in 12-well plates. After 10 to 14 days, the cells were fixed with 10% formaldehyde neutral buffer solution (Nacalai Tesque, Kyoto, Japan) and stained with rhodamine B (Wako, Osaka, Japan). Colony formation was assessed using a dissecting microscope and the CFE was calculated. Cell sheet generation was performed as previously reported^13^. Briefly, the cells isolated by cell sorting were seeded onto LN511E8-coated dishes at a density of 1 × 10^5^ cells/well in 12-well plates or in cell culture inserts and cultivated in CEM and corneal epithelium maturation medium (CMM; i.e. KCM medium containing 10–20 ng/mL KGF and 10 μM Y-27632) for 14–21 days.

### Serial cell passaging assay

CE lineage cells isolated by cell sorting were cultivated on MMC-treated NIH-3T3 feeder layers in CMM up to 70–80% confluence. The CECs were harvested using TrypLE Express (Life Technologies) following the removal of feeder cells by manual pipetting. Cells were counted and seeded onto newly prepared feeder layers at a cell density of 20000 cells per well in 12-well plates. These cells were cultivated in CMM until sub-confluence was once more reached.

### Immunofluorescence staining

Cells or tissues were fixed in 4% paraformaldehyde (PFA, Wako), washed with Tris-buffered saline (TBS, TaKaRa Bio, Shiga, Japan) three times for 10 min and incubated with TBS containing 5% donkey serum and 0.3% Triton X-100 for 1 h to block non-specific reactions. Afterwards, they were incubated with the antibodies listed in Supplementary Table S1 at 4 °C overnight or at room temperature for 3 h. The cells were again washed twice with TBS for 10 min, and incubated with AF488- and 568-conjugated secondary antibodies (1:200; Life Technologies) for 1 h at room temperature. For p40 staining, the slides were incubated with Biotin-SP-conjugated secondary antibody (1:200, Jackson ImmunoResearch Laboratories, West Grove, PA, USA) for 1 h at room temperature. After two washes with TBS for 10 min, the slides were incubated with AF488-conjugated Streptavidin (1:200, Jackson ImmunoResearch Laboratories) for 1 h at room temperature. Counterstaining was performed with Hoechst 33342 (Life Technologies) prior to fluorescence microscopy (Axio Observer.D1, Carl Zeiss, Jena, Germany). For CD200 immunostaining, the PE-conjugated antibody was directly added to the medium and the cells were incubated for 3 h at 37 °C. Research-grade human corneo-scleral tissue was obtained from Northwest Lions Eye Bank (Seattle, WA, USA). To examine mouse eyes, pregnant females (ICR, E12.5) were acquired from SLC Japan (Shizuoka, Japan). All animal experimentation was performed in accordance with the ARVO Statement for the Use of Animals in Ophthalmic and Vision Research, and was approved by the animal ethics committee of Osaka University.

### Gene expression analysis

Real-time quantitative PCR was performed using the ABI Prism 7500 Fast Sequence Detection System (Life Technologies) in accordance with the manufacturer’s instructions. The TaqMan probes (Life Technologies) used in the present study are listed in Supplementary Table S2. The thermocycling program was performed with an initial cycle at 95 °C for 20 s, followed by 45 cycles at 95 °C for 3 s and 60 °C for 30 s. For the screening of negative makers for CECs, microarray data deposited in Gene Expression Omnibus under the accession number GSE73971 were used.

### Single-cell gene expression analysis

Single-cell gene expression profiling was performed using the Fluidigm Biomark dynamic array (Fluidigm, San Francisco, CA, USA) according to the manufacturer’s instructions. Briefly, human iPSC-derived CEC sheets obtained by CD200-negative sorting were dissociated by Accutase, and the cells were stained with 7-AAD (BD Bioscience). 7-AAD-negative live cells were isolated with a FACSAriaII cell sorter and applied to C1 Single-Cell Auto Prep IFC (Fluidigm) to prepare single cells and amplify the target sequence. Real-time quantitative PCR analyses using the TaqMan probes were performed with BioMark HD system (Fluidigm). The thermocycling was performed using an initial cycle at 95 °C for 60 s, followed by 35 cycles at 95 °C for 5 s and 60 °C for 20 s. Data were analysed by Singular (Fluidigm) and JMP software programs (SAS Institute Inc., Cary, NC, USA).

### Statistical analysis

All data are expressed as means ± SD. Wilcoxon rank sum tests were performed to analyse the differences between two groups. All statistical analyses were performed with JMP software program. P-values less than 0.05 were considered statistically significant.

## Electronic supplementary material

Supplementary imformation
