# Supplementary material for: CD200 facilitates the isolation of corneal epithelial cells derived from human pluripotent stem cells
Source: Sci Rep. 2018 Nov 8;8:16550. doi: 10.1038/s41598-018-34845-2 (PMC6224558; doi:10.1038/s41598-018-34845-2)
Supplement: Supplementary file 1 — Supplementary imformation [file 41598_2018_34845_MOESM1_ESM.pdf]

# CD200 facilitates the isolation of corneal epithelial cells derived from human pluripotent stem cells

Ryuhei Hayashi[1,2*], Yuki Ishikawa[2], Tomohiko Katayama[2], Andrew J. Quantock[3], and Kohji Nishida[2*]

[1] Department of Stem Cells and Applied Medicine, Osaka University Graduate School of Medicine, Suita, Osaka, 565-0871, Japan. [2] Department of Ophthalmology, Osaka University Graduate School of Medicine, Suita, Osaka, 565-0871, Japan. [3] Structural Biophysics Group, School of Optometry and Vision Sciences, College of Biomedical and Life Sciences, Cardiff University, Cardiff, CF24 4HQ, Wales, UK.

*Corresponding to ryuhei.hayashi@ophthal.med.osaka-u.ac.jp and knishida@ophthal.med.osaka-u.ac.jp

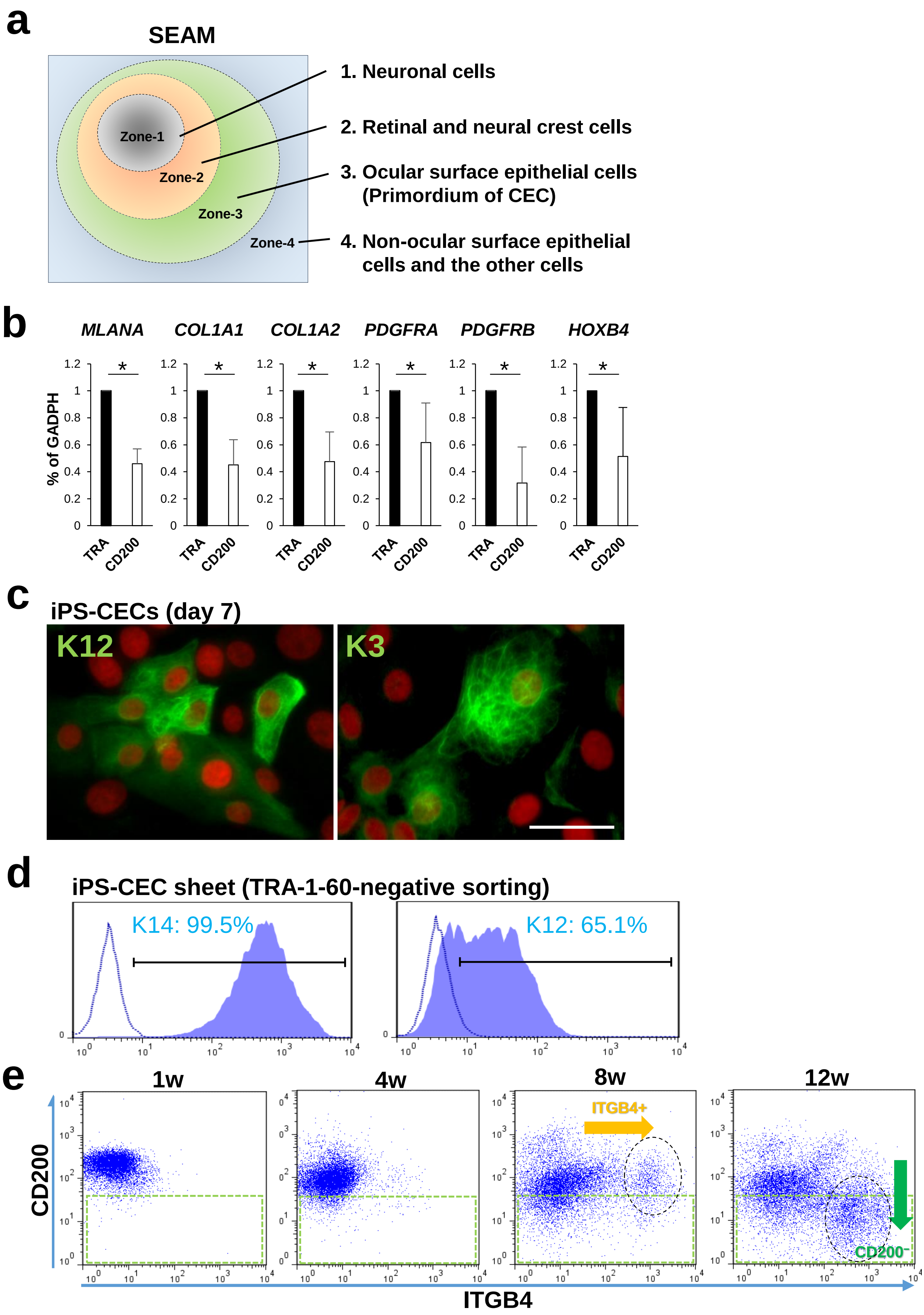


# Supplementary Figure S1

a

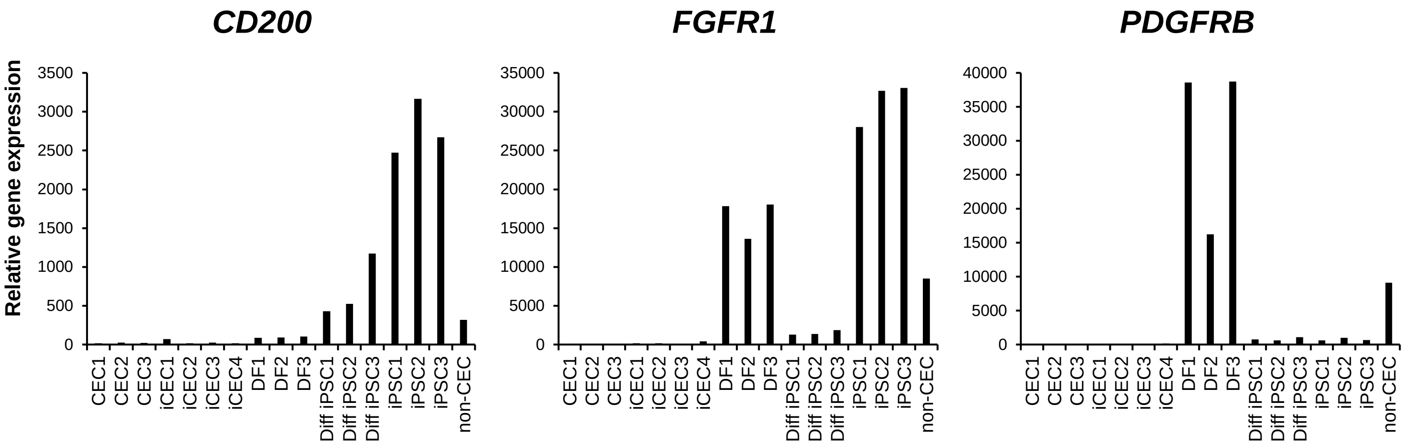


iCEC: iPSC-derived CECs,
DF: Dermal fibroblasts,
Diff iPSC: 6 weeks differentiated iPSCs
non-CEC: Non-CEC colonies

# Supplementary Figure S2

| Antigen | Description | |
|---|---|---|
| **K12** | Goat polyclonal; N-16 | Santa Cruz Biotechnology, Santa Cruz, CA, USA |
| **K3** | Mouse monoclonal; AE5 | PROGEN Biotechnik, Heidelberg, Germany |
| **MUC16** | Mouse monoclonal; OV185:1 | Abcam, Cambridge, MA, USA |
| **p63** | Mouse monoclonal; 4A4 | Santa Cruz Biotechnology |
| **p40 (delta-N p63)** | Rabbit polyclonal; API 3030 AA | Biocare Medical, Concord CA |
| **K14** | Rabbit polyclonal; Poly19053 | Biolegend |
| **K15** | Rabbit monoclonal; EPR1614Y | Abcam |
| **K4** | Mouse monoclonal; 6B10 | Abcam |
| **K13** | Mouse monoclonal; AE8 | Abcam |
| **Pan-cytokeratin (K5/K8)** | Mouse monoclonal; FITC-conjugated | PROGEN Biotechnik |
| **PAX6** | Rabbit polyclonal; PRB-278P | Covance Research Products, Denver, PA, USA |
| **CD200** | Mouse monoclonal; OX-104 PE-conjugated | Biolegend |
| **CD200** | Goat polyclonal; AF2724 (for mouse tissues) | R&D systems, Minneapolis, MN, USA |

# Supplementary Table S1

| Target gene | Assay ID |
|---|---|
| *GAPDH* | Hs99999905_m1 |
| *PAX6* | Hs00240871_m1 |
| *DN-p63* | Hs00978339_m1 |
| *K3* | Hs00365080_m1 |
| *K10* | Hs00166289_m1 |
| *K12* | Hs00165015_m1 |
| *K13* | Hs00999762_m1 |
| *K18* | Hs01941416_g1 |
| *CDH1* | Hs01023894_m1 |
| *CDH2* | Hs00983056_m1 |
| *CRYAA* | Hs00166138_m1 |
| *18S* | Hs99999901_s1 |
| *CD200* | Hs01033303_m1 |
| *POU5F1* | Hs00999632_g1 |
| *FGFR1* | Hs00915142_m1 |
| *SOX2* | Hs01053049_s1 |
| *SOX9* | Hs00165814_m1 |
| *RPE65* | Hs01071462_m1 |
| *TUBB3* | Hs00801390_s1 |
| *VSX2* | Hs01584047_m1 |
| *HOXA1* | Hs00939046_m1 |
| *HOXB4* | Hs00256884_m1 |
| *MLANA* | Hs00194133_m1 |
| *COL1A1* | Hs00164004_m1 |
| *COL1A2* | Hs01028956_m1 |
| *PDGFRA* | Hs00998018_m1 |
| *PDGFRB* | Hs00182163_m1 |
| *RUNX1* | Hs01021971_m1 |
| *AQP5* | Hs00387048_m1 |

# Supplementary Table S2
